# The Serpin Superfamily in Adipose Tissue Remodeling: Molecular Drivers of Immune–Metabolic Crosstalk and Insulin Sensitivity

**DOI:** 10.3390/biology15130989

**Published:** 2026-06-23

**Authors:** Nouran Alwisi, Alaa Abdelhamid, Amna Al-Quradaghi, Maha Talhami, Aldana M. Alkuwari, Nadia Alsharif, Jessica Saliba, Abdullah A. Shaito

**Affiliations:** 1 College of Medicine, QU Health, Qatar University, Doha P.O. Box 2713, Qatar; na2104730@qu.edu.qa (N.A.);; 2 Department of Biomedical Sciences, College of Health Sciences, QU Health, Qatar University, Doha P.O. Box 2713, Qatar; 3 Department of Public Health, Faculty of Health Sciences, University of Balamand, Sin El Fil P.O. Box 55251, Lebanon; 4 Biomedical Research Center (BRC), QU Health, Qatar University, Doha P.O. Box 2713, Qatar

**Keywords:** serpins, obesity, insulin resistance, ECM remodeling, immune–metabolic crosstalk, fibrosis, angiogenesis

## Abstract

Adipose tissue remodeling is a dynamic process essential for metabolic homeostasis, enabling tissue expansion, extracellular matrix (ECM) turnover, angiogenesis, and coordinated immune adaptation. In obesity, however, maladaptive remodeling characterized by fibrosis, chronic low-grade inflammation, and hypoxia disrupts adipose plasticity and promotes systemic insulin resistance. Central to these processes is the tightly regulated homeostasis between proteases and their inhibitors, in which the serine protease inhibitor (serpin) superfamily represents an important yet underappreciated regulatory axis. Beyond their classical roles in coagulation and fibrinolysis, serpins regulate ECM remodeling, macrophage recruitment and polarization, cytokine signaling, angiogenic responses, adipokine activity, and insulin sensitivity, thereby orchestrating immune–metabolic crosstalk within adipose depots. Emerging evidence indicates that individual serpins exert distinct and context-dependent effects, with some promoting fibrosis, inflammation, and metabolic dysfunction, whereas others preserve adipose tissue homeostasis and metabolic function. This review synthesizes current knowledge on the structural and functional diversity of the serpin superfamily and examines their mechanistic roles in adipose tissue remodeling during obesity, with particular emphasis on how adipose-associated serpins regulate adipose tissue homeostasis, depot-specific remodeling, and immune–metabolic crosstalk. The review further discusses the experimental and translational applications of emerging single-cell and spatial transcriptomics, multi-omics, and computational approaches that may advance the understanding of serpin biology, improve the investigation of human adipose tissue, and accelerate the identification of clinically relevant serpin-related biomarkers and therapeutic targets for obesity and related metabolic disorders. By positioning serpins as key regulators of adipose tissue remodeling and immune–metabolic integration, this review highlights protease–antiprotease balance as a central determinant of metabolic health and identifies serpins as promising biomarkers and therapeutic targets for obesity and related metabolic disorders.

## Simple Summary

This review summarizes current evidence on how serpins regulate adipose tissue function during obesity. Some serpins, such as plasminogen activator inhibitor-1 (PAI-1, SERPINE1), promote fibrosis, inflammation, and insulin resistance, whereas others, including kallistatin (SERPINA4), alpha-1 antitrypsin (SERPINA1), and vaspin (SERPINA12), help preserve healthy adipose tissue function and metabolic balance. We discuss how disturbances in protease–antiprotease (serpin) regulation contribute to adipose tissue remodeling and metabolic disease, and highlight emerging technologies that may facilitate the discovery of new serpin-related biomarkers and therapeutic targets for obesity and related metabolic disorders.

## 1. Introduction

Adipose tissue is no longer considered a passive location of fat storage. It is now appreciated as a dynamic immunometabolic organ, which plays a role in regulating energy balance, endocrine signaling, inflammation, and insulin sensitivity of the whole body [1]. Under normal circumstances, adipose tissue can grow and restructure in a regulated manner to allow excess nutrients to be stored without significant metabolic harm. This process involves adipocyte hypertrophy, precursor cell differentiation, extracellular matrix (ECM) remodeling, angiogenesis and direct contact of the tissue with immune system cells [2,3]. Immune–adipocyte crosstalk plays a critical role in maintaining tissue homeostasis, involving interactions with macrophages, T cells, and other immune populations that regulate inflammatory tone.

In contrast, adipose tissue remodeling in obesity is typically maladaptive. Instead of supporting healthy expansion, the tissue develops a state of chronic low-grade inflammation, inadequate vascular adaptation, progressive fibrotic extracellular matrix accumulation, and localized hypoxia. Collectively, these changes reduce adipose tissue plasticity and contribute to insulin resistance [3,4]. The expansion of pro-inflammatory macrophage populations and the dysregulated release of inflammatory cytokines constitute early and functionally critical events in dysfunctional adipose remodeling, driving impaired insulin signaling in adipocytes and other metabolic tissues [4].

The balance between protease activity and antiprotease regulation is a central process in adipose tissue remodeling. Proteases contribute to extracellular matrix degradation, cell migration, and tissue restructuring, whereas protease inhibitors limit excessive proteolysis and help preserve tissue integrity [1]. Within this regulatory network, the serpin superfamily has received growing attention because several of its members influence processes directly relevant to adipose biology, including fibrinolysis, matrix remodeling, immune-cell recruitment, angiogenesis, and inflammatory signaling [5]. Thus, serpins are recognized not only as classical protease inhibitors but also as key molecular regulators of the adipose tissue microenvironment [6,7].

The serpin superfamily comprises a large group of structurally related proteins that regulate protease activity across diverse biological systems. Most serpins share a conserved fold composed of three β-sheets, several α-helices, and a reactive center loop (RCL), which mediates interaction with target proteases [8]. Serpins inhibit serine proteases through a suicide-substrate mechanism. The target protease first recognizes and cleaves the exposed RCL, triggering a major conformational rearrangement in the serpin. The cleaved RCL inserts into β-sheet A, dragging the covalently linked protease and distorting its active site. This irreversibly traps the enzyme in an inactive serpin–protease complex.

Plasminogen activator inhibitor-1 (PAI-1/SERPINE1) is among the most extensively studied serpins in metabolic disease. Human and rodent studies identify adipose tissue as a major source of PAI-1, with its secretion markedly increased in obesity, linking adipose dysfunction to impaired fibrinolysis and a heightened inflammatory state [5]. Experimental obesity models further demonstrate that PAI-1 actively influences adipose cellularity and tissue expansion, suggesting an active role in remodeling rather than a purely biomarker function [2]. Elevated PAI-1 has also been associated with adipose inflammation and metabolic dysfunction that contribute to impaired insulin responsiveness, supporting its relevance to immunometabolic crosstalk in adipose tissue [9].

The review provides an overview of serpin structure and function, including their RCL-dependent suicide-substrate inhibitory mechanism, clade-based classification, and functions extending beyond protease inhibition, such as hormone transport and immune modulation. The review further examines how adipose tissue-relevant serpins contribute to obesity-associated adipose tissue remodeling and metabolic dysfunction by regulating ECM turnover, fibrosis, inflammation, angiogenesis, hypoxia responses, adipokine signaling, immune–metabolic crosstalk, and insulin sensitivity. Particular emphasis is placed on how protease–antiprotease imbalance promotes ECM accumulation, tissue stiffness, vascular maladaptation, impaired adipose expandability, chronic inflammation, and systemic insulin resistance during obesity. Throughout the review, evidence is discussed according to its experimental and translational context, including direct human adipose tissue studies, murine and other rodent models, non-adipose metabolic studies, and more inferential findings. This distinction is especially relevant for SERPINA3 and its murine homolog Serpina3c, for which metabolic and inflammatory functions have been primarily described in mouse adipose tissue models [10], whereas human evidence remains largely indirect and derived from non-adipose inflammatory contexts, including liver and plasma studies [11]. Finally, the review highlights emerging omics approaches, including single-cell and spatial transcriptomics, as promising tools for identifying adipose-specific serpin signatures and potential therapeutic targets in metabolic disease.

## 2. The Serpin Superfamily: Structure, Classification, and Functional Diversity

### 2.1. Structural Basis of Serpin Inhibition

**Figure 1:**
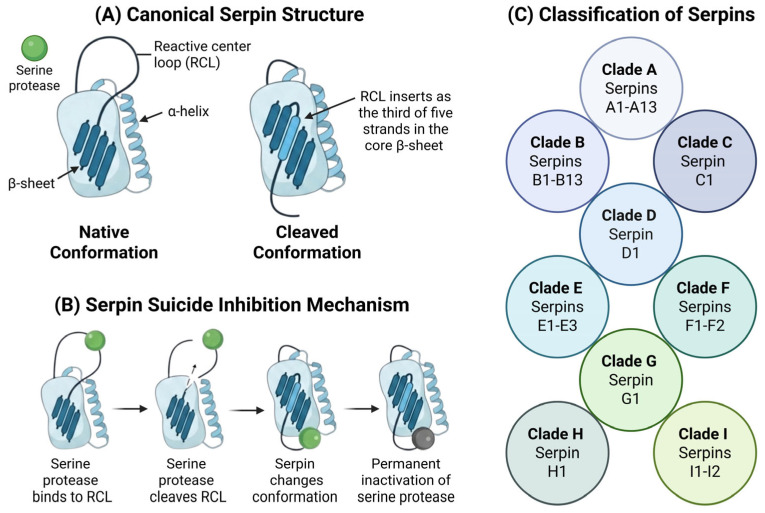
The Serpin Superfamily: Structure, Mechanism of Action, and Classification. (**A**) The canonical serpin structure in its native conformation consists of α-helices, β-sheets, and a reactive center loop (RCL). Upon RCL cleavage by serine proteases, the serpin undergoes a conformational change where the RCL inserts into the β-sheet as the third of five strands. (**B**) Serpins are serine protease inhibitors that act as suicide inhibitors by employing a “molecular mousetrap” mechanism. The serine protease cleaves the serpin’s RCL, inducing rapid serpin conformational change. This transition inserts the RCL into the serpin’s β-sheet whilst simultaneously carrying the attached protease and deforming its active site, leading to irreversible inactivation of the serine protease. (**C**) The serpin superfamily in humans consists of 37 members classified into nine main clades (A–I). Created with https://www.biorender.com accessed on 8 April 2026.

A hallmark of serpins is the RCL, which contains a protein recognition site connecting β sheets A and C through a flexible stretch of residues [12]. The RCL consists of approximately 20 residues and serves as a “pseudosubstrate” for the target protease [13,14]. In addition, RCL structure and β-sheet play a crucial role in mediating the functions of serpins through a conformational change from stressed to relaxed [15,16,17]. To elaborate, RCL cleaves and a conformation change occurs, which results in the insertion of RCL into β-sheet A [16,18]. This is referred to as the suicide inhibition mechanism, as the conformational change inhibits the protease irreversibly and therefore regulates proteolytic processes (Figure 1) [17,19]. But inhibition can also occur reversibly in cytotoxic lymphocytes. A study shows that reactive oxygen species (ROS) inactivate SERPINB9 through the formation of a vicinal disulfide bond in the RCL, which then inhibits enzyme granzyme B (GzmB)-mediated death and protects lymphocytes as a result. This means that once ROS levels decrease, SERPINB9 will become functional again [20].

At a structural level, it could be observed that serpin has both conserved and non-conserved regions across the serpin family. Phylogenetic analysis reveals that the RCL is the fastest evolving region within the serpin nucleotide sequence. Protein sequence analysis demonstrates that the specific P1 residue within the RCL determines which target proteinases an inhibitory serpin can trap [15]. Along with this rapid evolving region, conserved regions can also be observed in the RCL. An evolutionary analysis found that every inhibitory serpin contains a highly conserved hinge region within its RCL [21]. An example of this would be in SERPINA3 where a subgroup of six bovine SERPINA3 members was discovered to have an unexpectedly high degree of sequence conservation with more than 82% identity for the RCL domain. The study identified a specific arginine residue as the likely P1 site for trypsin cleavage in bovine SERPINA3-1 through SERPINA3-6 [22]. In addition, studies have identified the P14 position (Ser-380) as a crucial prerequisite for inhibitory activity. The study determined that approximately −32 kcal/mol of energy is released when the RCL is incorporated into the main β-sheet. The research hypothesized that helix B exposure in serpin variants increases the propensity for polymerization by interfering with smooth RCL translocation [18,23].

### 2.2. Classification of Serpins

Serpins represent one of the largest superfamilies of protease inhibitors identified across all kingdoms of life [19]. In confirmation, Huntington’s review reported over 1500 serpin sequences across all kingdoms, with up to 37 serpins being described in humans [24]. Across species, serpins are divided into 16 Clades (A–P), whereas the 37 human serpins belong to nine Clades (A–I), with Clades A and B representing the largest groups within human serpins [15,19]. Human clades A and B encompass 13 members, Clade E has 3 members, Clades F and I have 2 members, and Clades C, D, G, and H each consists of a single serpin member. Each serpin is named SERPINXY, in which X represents the clade and Y is the number within the clade [19,25]. The human serpins within Clades A-I exhibit marked diversity in subcellular localization, genomic organization, and biological function. Clade A comprises extracellular serpins encoded on chromosomes 1, 14, and X and includes both inhibitory and non-inhibitory members [16]. Representative inhibitory proteins include SERPINA1 (α1-antitrypsin) and SERPINA3 (α1-antichymotrypsin), whereas SERPINA6 and SERPINA7 function as corticosteroid- and thyroxine-binding globulins, respectively [16]. In contrast, clade B (ov-serpins) consists of 13 predominantly intracellular proteins encoded on chromosomes 6 and 18 that lack signal peptides and possess shorter terminal regions [16,21]. This clade encompasses inhibitors of serine and papain-like cysteine proteases, including SERPINB3, SERPINB6, and SERPINB9, as well as the non-inhibitory SERPINB5 (maspin), a recognized suppressor of tumor progression [16,26].

The remaining clades comprise one or a few highly specialized members with distinct physiological functions. SERPINC1 (Clade C) and SERPIND1 (Clade D) regulate coagulation through inhibition of thrombin and related proteases, whereas clade E members, including SERPINE1 (PAI-1), SERPINE2, and SERPINE3, primarily modulate fibrinolysis [16]. Clade F contains the anti-angiogenic and neurotrophic SERPINF1 (PEDF) together with the plasmin inhibitor SERPINF2 (α2-antiplasmin), while SERPING1 (Clade G) is the principal regulator of the classical complement pathway. Clade H is represented by the collagen-specific chaperone SERPINH1 (HSP47), which lacks protease inhibitory activity, whereas Clade I comprises the neurotrophic SERPINI1 (neuroserpin) and SERPINI2 (pancipin), the latter having been implicated in suppression of tumor metastasis [16].

### 2.3. Phylogenetic Evolution and Structural Basis of Serpin Function

The evolutionary history of the 37 human serpins reflects a balance between structural conservation and functional diversification. Phylogenetic analyses based on large-scale structural alignments indicate that the serpin superfamily is organized around a conserved central core from which specialized clades have evolved [27]. Intracellular clade B serpins are considered ancestral to most extracellular clades, whereas SERPINC1 (antithrombin, clade C) occupies an intermediate position linking intracellular and extracellular serpins, suggesting an early transition toward extracellular functions [27,28]. In contrast, highly specialized members such as SERPINH1 (HSP47), which functions as a collagen chaperone rather than a protease inhibitor, are among the most evolutionarily divergent [27]. Phylogenetic relationships broadly parallel genomic organization, and available evidence suggests that serpin diversification was driven primarily by gene duplication and speciation rather than co-evolution with target proteases [21].

### 2.4. Reactive Center Loop Dynamics and Functional Diversity of Serpins

Although most serpins inhibit serine proteases, some display cross-class activity against cysteine proteases, including SERPINB3 and SERPINB13 [29]. Others, such as ovalbumin, have lost inhibitory capacity because their reactive center loop (RCL) cannot efficiently undergo the conformational changes required for protease trapping [24]. Despite their functional diversity, human serpins share a highly conserved structural framework consisting of three β-sheets, seven to nine α-helices, and a mobile RCL while exhibiting substantial sequence divergence [21,30]. A conserved shutter domain, particularly residues Ser53 and Ser56, is thought to maintain structural stability and the conformational flexibility required for RCL-mediated function [21,31].

RCL is the primary determinant of whether the serpin will be inhibitory or non-inhibitory [32]. This can be observed in a genome-wide analysis of the codling moth (*Cydia pomonella*), which provided a comprehensive set of serpin genes, each possessing a different RCL sequence and therefore functionality. For instance, CpSPN22 and CpSPN26 were reported to have a high chance of being non-inhibitory serpins. This is due to the deviated length of their RCL, where CpSPN22 lacked an RCL region and CpSPN26 contained an insertion mutation that resulted in a longer RCL [33]. Although the study was not done on humans, we can still conclude that the length of RCL could influence the way it functions. However, the length of the RCL alone cannot suffice; its amino acid profile also needs to be considered. A functional analysis study identified residues of positions 17 to 9 to be crucial for the inhibitory function. The study provides the consensus pattern for this region in inhibitory serpins, which is: P17[E]-P16[E/K/R]-P15[G]-P14[T/S]-P12-P9[A/G/S] [4]. Based on the pattern, serine or threonine is at P14, while glycine is at P15, and positions 12 to 9 have short side-chain residues. These residues facilitate efficient insertion of the RCL during protease inhibition, and mutations in these crucial regions convert an inhibitory serpin to a non-inhibitory one [28,34].

Although the RCL sequence may provide partial indication of the functional outcome, local electrostatics and RCL dynamics dictate the rate of insertion during protease inhibition, and therefore whether it behaves as an inhibitor or a substrate. If RCL insertion is swift, the inhibitory pathway progresses, and as a result, the protease is trapped. On the other hand, if insertion is lacking speed, the protease de-acylates and escapes, which causes the serpin to act as a non-inhibitory substrate. Reasons for slow rates of insertion are increased dynamics in the hinge region or the displacement of the hinge away from the β-sheet, which causes the behavior of an inhibitor to shift toward a substrate-like one [35]. Moreover, the flexibility of the RCL dictates whether a serpin can successfully fit a target protease active site. A non-inhibitory serpin ovalbumin has a rigid RCL with a fully extended alpha-helical conformation, while an inhibitory serpin RCL is more flexible. Therefore, a lack of inhibitory activity can be observed in the rigid RCL as it fails to confirm the protease active site [18]. This could be explained through evolution, as various members have lost this inhibitory ability by alteration of RCL and have instead acquired new roles, for instance, hormone carriers such as cortisol-binding globulin (CBG) and thyroxine-binding globulin (TBG), or as hormone precursors like angiotensinogen [15]. Non-inhibitory serpins can adapt the RCL’s structural transition for regulated cargo release rather than enzyme inhibition. In CBG, the cleavage of the RCL by neutrophil elastase at inflammatory sites induces an S-to-R transition, changing the serpin’s conformation. This change leads to a tenfold decrease in affinity for its ligand, resulting in the direct release of cortisol at the site of inflammation [36].

### 2.5. Beyond Protease Inhibition

As mentioned before, the largest clades in human serpins are clade A and clade B, each differing in chromosome location and putative signal peptides that determine whether they are extracellular or intracellular. Clade A serpins are extracellular, located on chromosomes 14 and X, and possess putative signal peptides, whereas clade B serpins are intracellular, located on chromosomes 6 and 18, and lack putative signal peptides [16,19,25,37,38]. These differences influence how each type carries out different and specific functions beyond protease inhibition. A phylogenetic analysis concluded that extracellular serpins are involved in multicellular processes like blood coagulation and hormone delivery. Conversely, intracellular serpins have been reported to function in processes such as granule-mediated apoptosis [28]. In addition, a study reports intracellular clade B serpins to function as regulators of inflammation and angiogenesis [14].

It is also noteworthy to mention serpin’s signaling function in modulating intracellular signaling, immune modulation and homeostasis. Recent work revealed a previously unknown Serpina3c/Cathepsin G/Integrin/AKT pathway in mouse adipose tissue. The study found that the secreted factor Serpina3c inhibits Cathepsin G activity, which blocks the turnover of α5 and β1 integrins. This thereby induces intracellular AKT activity to suppress JNK and inhibit inflammation [10]. Another anti-inflammatory discovery was reported in Rezaie and Giri’s study. The study detects an anti-inflammatory signaling function of a type of serpin called Antithrombin (AT), mediated by its binding to Syndecan-4 (Synd-4) on vascular endothelial cells. It has been indicated that native AT recruits Protein Kinase C delta (PKC-δ) to the cytoplasmic membrane. This recruitment induces prostacyclin (PGI2) synthesis and inhibits nuclear factor κ B (NF-κB). Alternatively, a cleaved or latent form of AT links with perinuclear/nuclear localization of PKC-δ to activate proapoptotic pathways [39]. In addition, serpins’ roles in regulating innate immune pathways like the Toll pathway and maintaining immune homeostasis can be observed. Studies done on *Drosophila* report serpins’ role in regulating innate immune cascades like the Toll and prophenoloxidase pathways [40]. Serpin43Ac in particular neutralizes Persephone and therefore negatively regulates the Toll pathway [41]. Additionally, Serpin1A and Serpin6 synergistically maintain silkworm immune homeostasis by inhibiting an extracellular terminal protease, CLIP2. It was found that CLIP2 processes the ligand proSpätzle to activate the Toll signaling pathway and induce antimicrobial peptide expression [42]. Importantly, serpin regulation of Toll signaling has been best characterized in invertebrates, particularly Drosophila, where serpins modulate extracellular protease cascades upstream of Toll receptor activation rather than directly interacting with the receptor itself. In this pathway, proteolytic activation of the cytokine-like ligand Spätzle triggers Toll signaling through MyD88, Tube, and Pelle, culminating in nuclear translocation of the NF-κB-related transcription factors Dorsal and Dif. Serpins such as Serpin43Ac negatively regulate this process by inhibiting upstream proteases, including Persephone, thereby limiting excessive innate immune activation [41].

Direct regulation of mammalian Toll-like receptors (TLRs) by serpins has not been established; however, several serpins modulate TLR-associated inflammatory responses indirectly. For example, SERPINA1 suppresses TLR4-dependent NF-κB activation and cytokine production in macrophages and endothelial cells, whereas PAI-1 (SERPINE1) and PEDF (SERPINF1) can amplify inflammatory responses through interactions with NF-κB, JNK, and related cytokine networks [43]. These findings suggest that serpins influence innate immunity primarily by regulating extracellular proteases and downstream inflammatory pathways rather than through direct receptor binding.

The functional consequences of these interactions vary considerably among family members. While some serpins exert anti-inflammatory and metabolically protective effects, others promote adipose inflammation, extracellular matrix remodeling, macrophage recruitment, fibrosis, and insulin resistance. In particular, PAI-1 (SERPINE1), PEDF (SERPINF1), and SERPINA3 have been implicated in obesity-associated adipose tissue dysfunction, highlighting the context-dependent and functionally diverse roles of the serpin superfamily in adipose biology.

## 3. Protease–Antiprotease Networks in Adipose Tissue

### 3.1. ECM Turnover and the Plasminogen Activation System

Serpins act as important regulators of protease activity during ECM remodeling. A deficiency in serpins can lead to disrupted fibrinolysis, along with matrix turnover. This drives observations like impaired wound closure and hindered re-epithelialization in diabetic tissue [44]. One of the vital contributors to such a mechanism is PAI-1, also known as SERPINE1. PAI-1 has been identified as a central player in ECM remodeling via the uPA/uPAR system. Through molecular events such as protein translocation and enzyme catalysis, PAI-1 contributes to structural integrity of the extracellular environment [45,46]. A prior study done on human mesangial cells showed that the inhibition of PAI-1 with monoclonal antibodies yielded a four-fold increase in ECM degradation. Without the inhibition of PAI-1, the usual cascade of events is initiated by either uPA or tPA, which contributes to the production of plasmin from plasminogen [47]. Plasmin then activates matrix metalloproteinase (MMP)-2, along with MMP3, 9, 12, and 13 depending on the cell type, which directly degrades the ECM and releases growth factors [47]. From these results, PA-1 contribution is evident, where it is believed to be a negative regulator of the ECM degradation system [48].

**Figure 2:**
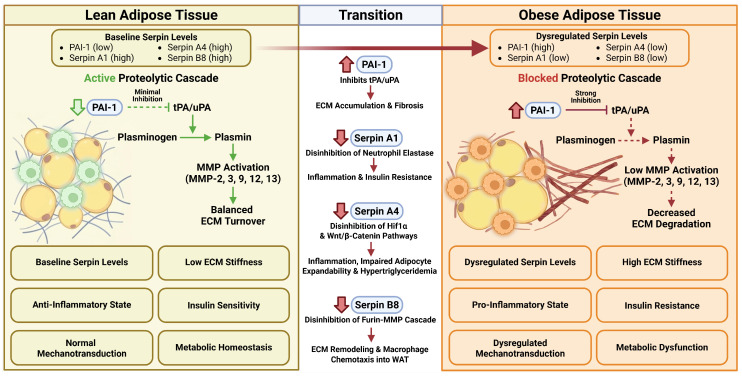
Protease–Antiprotease Network in Adipose Tissue Remodeling. In lean adipose tissue, low plasminogen activator inhibitor-1 (PAI-1) and high Serpin A1, Serpin A4, and Serpin B8 expression preserve protease–antiprotease balance. This supports tPA/uPA-mediated plasmin generation, MMP activation, balanced ECM turnover, low matrix stiffness, normal mechanotransduction, and insulin sensitivity. In obesity, elevated PAI-1 and reduced Serpin A1, Serpin A4, and Serpin B8 disrupt this balance. PAI-1 inhibits tPA/uPA activity, limiting plasmin formation and MMP-mediated ECM degradation, while reduced protective serpins promote neutrophil elastase activity, HIF-1α and Wnt/β-catenin signaling, and furin-MMP activation. Together, these changes drive ECM accumulation, fibrosis, inflammation, impaired adipocyte expandability, altered mechanotransduction, and insulin resistance. Created with https://www.biorender.com accessed on 8 April 2026.

Similarly, PAI-1 is also relevant in adipose tissue because it regulates ECM turnover through its effects on the plasminogen activation system. By inhibiting tPA and uPA, PAI-1 reduces plasmin formation, which is needed to activate latent MMPs that normally degrade ECM components. As a result, excessive or sustained PAI-1 activity decreases pericellular proteolysis and matrix degradation, leading to collagen accumulation, fibrosis, and impaired tissue remodeling (Figure 2). PAI-1 also influences ECM architecture by interacting with vitronectin and the uPAR on cell surfaces. Through competition with integrins and uPAR for vitronectin-binding sites, PAI-1 can promote cell detachment from the ECM and regulate cell migration. In pathological states such as diabetes, this inhibition of fibrinolysis and ECM turnover may contribute to microvessel abnormalities and abnormal matrix accumulation [49]. Another serpin named Serpina3n also plays a critical role in regulating ECM organization and fibrosis. A deficiency of Serpina3n has been reported to result in increased ECM turnover, elevated fibrosis, and a poorly compacted matrix. This could be explained through the unchecked activity of substrate proteases such as granzyme B [50].

### 3.2. Dysregulation in Obesity

ECM remodeling, reorganization, and adipose tissue expansion are characteristics of obesity. These traits are crucial to provide sufficient space for enlargement of adipocytes (hypertrophy) and to form subsequent ones from precursor cells through adipogenesis (hyperplasia) [51]. In addition, elastin deposition is increased in visceral fat depots with obesity, forming a mesh-like net that becomes denser. Denser collagen/elastin matrices could restrain the expansion of adipocytes, leading to hypertrophy and metabolic dysfunction [52]. This is relevant as an impaired expandability causes cell death through necrosis or apoptosis, attracting proinflammatory macrophages [51].

As previously mentioned, serpins have been shown in multiple studies to be involved in maintaining homeostasis. Therefore, their dysregulation can further exacerbate obesity symptoms. This is evident in SerpinA1, which is a hepatic factor required for healthy adipose tissue development, and its dysregulation can increase the likelihood of obesity. It was established that *serpinA1* knockout mice exhibited adipocyte hypertrophy, while SerpinA1 treatment induces the proliferation of preadipocytes [53]. It can thus be predicted that, in obesity, SerpinA1 levels are decreased, as reported in a study done in leptin-deficient obese mice [54]. Through glycoprotein enrichment and quantitative proteomics techniques, SerpinA1 expression levels were found to be significantly reduced in obese mice. Consequently, neutrophil elastase (NE), was found to be elevated significantly. The study concluded that the imbalance between SerpinA1 and NE is a key driver of obesity and its related inflammation and insulin resistance (Figure 2) [54]. A recent study identifies SerpinA1 as a regulator of adipose regeneration, thermogenesis, and systemic energy metabolism [53]. Hepatic-derived SerpinA1 promotes white and brown adipose tissue recovery in lipodystrophy models and enhances UCP1 expression, mitochondrial activity, adipose browning, energy expenditure, glucose tolerance, and cold-induced thermoregulation [53]. Conversely, SerpinA1 deficiency impairs mitochondrial function, thermogenesis, UCP1 expression, adipose recovery, and metabolic adaptation during high-fat feeding. Mechanistically, these effects may involve SerpinA1 interaction with EphB2 and downstream activation of p38, FAK, and Erk1/2 signaling pathways [53]. Another crucial player is SerpinA3N, which is suggested to play a role in obesity and dietary excess. A recent study in mice concluded that SerpinA3N negatively regulates leptin signaling; knocking out the *serpinA3N* gene in leptin-sensitive neurons reduces adiposity and weight gain in diet-induced obese mice [55].

A substantial body of research has focused on the role of serpins in adipose tissue differentiation and obesity (Table 1), particularly SERPINA4, Serpina3c and SERPINA3. Notably, investigators have employed Serpina3c in murine models to examine the functions and mechanisms of SERPINA3 [56,57]. In studies using Serpina3c, it has been discovered that *serpina3c* gene knockout in mice led to an impaired expandability of adipocytes, which decreased the white adipose tissue weight, triglyceride overflow, hypertriglyceridemia, and macrophage-mediated metaflammation. In normal conditions, Serpina3c regulates adipogenesis through the inhibition of the Wnt/β-catenin pathway and hypoxia-inducible factor (Hif)1α-glycolysis axis in adipocytes, as shown in mice models [11,57]. In obese patients, SERPINA3, which is Serpinea3c ortholog in humans, has shown elevated expression. The reason why such an increase occurs is due to the obesity-related overexpression of proinflammatory cytokine TNF-α. Mechanistically, TNF-α regulates SERPINA3 expression primarily through activation of the NF-κB and mitogen-activated protein kinase (MAPK) pathways. Binding of TNF-α to TNF receptor-1 (TNFR1) recruits adaptor proteins such as TRADD and TRAF2, leading to activation of the IKK complex and MAPK cascades, including JNK and p38. Subsequent NF-κB nuclear translocation enhances transcription of inflammatory genes, including SERPINA3, suggesting that chronic TNF-α signaling may contribute to increased SERPINA3 expression during obesity-associated adipose inflammation [58].

Conversely, SERPINA3 and its murine ortholog Serpina3c may influence inflammatory and metabolic responses through inhibition of proteases such as cathepsin G [59]. By limiting cathepsin G activity, Serpina3c has been linked to modulation of ERK, AKT, and NF-κB pathways involved in adipocyte differentiation, extracellular matrix remodeling, and inflammation [10]. Collectively, these findings support a reciprocal relationship in which TNF-α induces SERPINA3 expression, whereas SERPINA3 may modulate downstream inflammatory and metabolic processes.

The antiadipogenic effects of TNF-α are modulated by SERPINA3, which inhibits the typical differentiation of mature and functional adipocytes from preadipocytes. In contrast, another study suggested that Serpina3c promotes adipogenic differentiation. The proposed mechanism entails that Serpina3c inhibits cathepsin G, which in usual cases degrades integrin α5. This integrin facilitates the activation of pathways like Extracellular Signal-Regulated Kinase (ERK) and Protein Kinase B (PKB), also known as AKT pathways, necessary for adipogenesis [60]. Although the findings appear to be contradictory, it is important to consider that these differences may be due to the different experimental models used. In the study reporting inhibition of adipogenesis differentiation, human subjects were examined. Alternatively, the contrasting study used a mouse model and a structurally homologous, but not identical, serpin named Serpina3c [59]. Notably, the mouse study also acknowledged this as a limitation to the findings, underscoring the need to confirm whether Serpina3c findings in mice are comparable to SERPINA3 findings in humans [60]. Therefore, findings from the human study may provide a more representative indication of the processes occurring in the human body compared with those derived from mouse models.

Alongside SERPINA3/Serpina3c, SERPINA4 has emerged as another serpin with evidence linking it to adipose tissue dysfunction in obesity. SERPINA4 inhibits Hif1α binding to nuclear factor erythroid 2-related factor 2 (Nrf2), a transcription factor that regulates Hif1α [11]. In obesity, however, SERPINA4 levels are reduced; therefore, the Wnt/β-catenin pathway and Hif1α are activated (Figure 2) [61]. The Hif1α-glycolysis axis leads to de novo lipogenesis and hypertrophy rather than healthy hyperplasia [11]. The Wnt/β-catenin pathway inhibits the Peroxisome Proliferator-Activated Receptor γ (PPARγ) transcription factor related to adipose differentiation and lipolysis, which causes impaired adipose differentiation and function as a result [57]. SERPINA4 was demonstrated to have adipose tissue inflammation and oxidative stress inhibition capabilities [62]. A case–control study revealed that SERPINA4 is involved in reducing the expression of inflammation-related genes like C-C motif chemokine ligand 2, interleukin (IL)-1B, IL6, IL8, TNFA, and transforming growth factor β, and upregulating the expression of critical inflammatory inhibitors like Adiponectin, C1Q and collagen domain-containing (ADIPOQ) and Krüppel-like factor 4 (KLF4). In addition, SERPINA4 reduces oxidative stress by inhibiting NOX2 and HIF1A expression while promoting the expression of SIRT1 and FOXO1. Therefore, the serpin reduction seen in obesity leads to the development of obesity-associated comorbidities [62]. SERPINA4’s role can also be explained through its ability to inhibit Hif1α, where SERPINA4 depletion drives inflammatory M1 macrophages through the release of pro-inflammatory cytokines in an HIF-1α-dependent pathway [63,64]. Emerging evidence further suggests that SerpinB2 contributes to adipose tissue immune homeostasis by supporting the survival of resident anti-inflammatory macrophages. Reduced SerpinB2 expression during obesity may compromise macrophage viability, thereby promoting unresolved adipose inflammation, metaflammation, and progression toward insulin resistance and type 2 diabetes [65]. Another serpin that regulates inflammation associated with obesity is SERPINB8, more specifically, its relation to its target inhibitor, furin. It was discovered that the downregulation of SERPINB8 promotes activation of furin, which then activates the furin–MMP activation cascade. The cascade is crucial for ECM remodeling and macrophage chemotaxis into white adipose tissue (Figure 2) [66].

**Table 1:** Functional Roles and Molecular Targets of Serpin Family Members in Adipose Tissue Remodeling and Metabolic Regulation.

Serpin Name	Target Molecule/Pathway	Effect	Major Organ(s)/Cell Type(s) of Expression	Reference
PAI-1 (SERPINE1)	uPA and tPA; lipid metabolism genes (e.g., Pcsk9, Fgf21)	Deficiency leads to: ↑ four-fold ECM degradation. ↓ Wound closure. ↓ Structural integrity in the extracellular environment. ↑ (Elevation) leads to: ↑ ECM stiffness and fibrosis ↑ Liver steatosis and fibrosis ↑ Insulin resistance ↑ Pro-fibrotic and lipotoxic microenvironment.	Adipocytes, stromal vascular cells, macrophages, endothelial cells, liver	[44,45,47,48,67]
Serpina3c	Cathepsin G; Wnt/β-catenin signaling; HIF-1α–glycolysis axis	Deficiency leads to: ↑ Impaired adipogenesis ↑ HIF-1α–driven hypoxic/metabolic stress ↑ Adipose inflammation and reduced expandability ↑ (Elevation) leads to: ↑ Promotes adipocyte differentiation and healthy adipogenesis ↓ HIF-1α signaling and glycolytic reprogramming ↓ Wnt/β-catenin activity ↑ Improved adipose tissue function and metabolic homeostasis	White and brown adipocytes, cardiac tissue, brain, lung.	[59,68]
Serpina3n	Granzyme B and leptin signaling.	Deficiency leads to: ↑ ECM turnover. ↑ Fibrosis. ↑ Weight gain. ↑ Adiposity.	Hepatocytes, hypothalamic leptin-responsive neurons, osteoblasts, brain microglia.	[50,55]
SERPINA1	NE	Deficiency leads to: ↑ Adipocyte hypertrophy. ↑ NE activity. ↑ Metabolic dysfunction.	Primarily hepatocytes; secondary expression in macrophages, adipocytes, and endothelial cells.	[53,54]
Kallistatin (SERPINA4)	Wnt/β-catenin and Hif1α-glycolysis pathways. Inflammatory cytokines (TNF-α, LPS); NOX2, HIF1a; SIRT1, FOXO1	Aids in weight control. Deficiency leads to: ↓ Levels are found in obesity. ↑ Impaired adipocyte expandability. ↑ Hypertriglyceridemia ↑ macrophage-mediated metaflammation. ↑ (Elevation) leads to: ↓ Inflammatory cytokine expression. ↑ Anti-inflammatory gene expression (ADIPOQ, KLF4). ↓ Oxidative stress (↓NOX2, HIF1a; ↑SIRT1, FOXO1).	Liver, vascular endothelial cells, adipose tissue.	[62]
SERPINA3	Modulated by TNF-α. Cathepsin G → ERK and AKT pathways.	↑ Differentiation of functional adipocytes from preadipocytes.	Liver, adipocytes, macrophages, inflammatory tissues.	[11,57,60]
SERPINB8	Intracellular furin inhibitor	Triggers a furin–MMP cascade. ↑ ECM remodeling. ↑ Macrophage chemotaxis into fat tissue.	Platelets, keratinocytes, epithelial cells, adipose tissue.	[66]
Vaspin (SERPINA12)	Kallikrein 7 (KLK7); GRP78; LRP1 receptor; Neuropeptide Y (NPY)/POMC	↑ (Elevation) leads to: ↓ KLK7 activity → ↓ insulin degradation & ↓ macrophage infiltration. ↓ Endoplasmic reticulum stress via GRP78 binding. ↓ Hepatic glucose production (via vagus nerve). ↓ Food intake (↓NPY, ↑POMC in hypothalamus).	Visceral adipose tissue (adipocytes), skin keratinocytes, gastric mucosa (stomach).	[69,70,71,72,73,74,75,76,77,78]
PEDF (SERPINF1)	JNK and IKK/NF-κB pathways; IRS-1	↑ (Elevation) leads to: ↑ Activation of JNK and IKK. ↑ Serine phosphorylation of IRS-1, disrupting insulin signaling. ↑ Free fatty acid mobilization and lipolysis. ↑ Hepatic gluconeogenesis. ↑ Insulin resistance	Adipocytes, liver, retinal pigment epithelial cells, endothelial cells.	[6,79,80,81]

ADIPOQ: Adiponectin, C1Q and collagen domain-containing; AKT: Protein Kinase B; ERK: Extracellular Signal-Regulated Kinase; FOXO: Forkhead box O; GRP78: Glucose-Regulated Protein 78; HIF1α: Hypoxia-Inducible Factor 1 alpha; IKK: IκB Kinase (Inhibitor of κB Kinase); IRS-1: Insulin Receptor Substrate 1; JNK: c-Jun N-terminal Kinase; KLF4: Krüppel-Like Factor 4; LPS: lipopolysaccharide; MMP: Matrix Metalloproteinase; NE: Neutrophil Elastase; NF-κB: Nuclear Factor kappa-light-chain-enhancer of activated B cells; NOX2: NADPH Oxidase 2; NPY: Neuropeptide Y; PAI-1: Plasminogen Activator Inhibitor 1; POMC: Proopiomelanocortin; SIRT1: Sirtuin 1; TNF-α: Tumor Necrosis Factor alpha; tPA: Tissue Plasminogen Activator; uPA: Urokinase-type Plasminogen Activator. Arrows indicate direction of change: ↑ denotes increase or upregulation, and ↓ denotes decrease or downregulation.

## 4. Serpin Expression and Regulation in Adipose Depots

Adipose tissue is recognized as a dynamic endocrine and immunometabolic organ that secretes a wide range of bioactive mediators, including adipokines, cytokines, and regulators of proteolytic pathways that influence metabolic homeostasis, systemic energy balance through appetite-related signaling from the central nervous system (CNS), and tissue remodeling [82]. Among these mediators, members of the serpin (serine protease inhibitor) superfamily play key roles in the control of extracellular proteolysis and fibrinolytic activity through inhibition of target serine proteases [83]. One of the most extensively studied adipose-derived serpins is plasminogen activator inhibitor-1 (PAI-1), the principal physiological inhibitor of tissue-type and urokinase-type plasminogen activators (tPA and uPA), thereby regulating plasmin generation and downstream extracellular matrix turnover [83,84]. Experimental studies have demonstrated that adipocytes express and secrete PAI-1, indicating that adipose tissue represents an important peripheral source of circulating PAI-1 [85,86]. In addition to mature adipocytes, several other cell populations within adipose depots, including stromal vascular cells, endothelial cells, and infiltrating macrophages, also contribute to local serpin production, reflecting the complex cellular regulation of protease inhibition within adipose tissue [84]. Importantly, serpin expression, particularly plasminogen activator inhibitor-1 (PAI-1), is markedly elevated in obesity and insulin-resistant states, linking adipose serpin dysregulation to metabolic disturbances and increased cardiometabolic risk [9,85]. These observations highlight the importance of understanding how serpin expression is regulated across different adipose tissue depots.

### 4.1. White Adipose Tissue (WAT) 

White adipose tissue (WAT) represents the primary site of energy storage in mammals, storing excess energy in the form of triglycerides and contributing to the regulation of systemic metabolic homeostasis [87]. In addition to its role in lipid storage, WAT contributes to metabolic regulation and tissue remodeling through the secretion of multiple bioactive mediators [82]. Within this context, serpins such as plasminogen activator inhibitor-1 (PAI-1) are expressed and secreted as part of the adipose tissue secretome, where they regulate proteolytic activity and extracellular matrix turnover within adipose depots [88]. As adipose tissue depots differ substantially in their cellular composition, metabolic activity, and inflammatory status, the expression and regulation of serpins may also vary between distinct adipose compartments [88].

WAT is broadly divided into visceral adipose tissue (VAT) and subcutaneous adipose tissue (SAT), two depots that differ substantially in their metabolic activity and inflammatory profile [87]. Visceral adipose tissue is generally regarded as more metabolically active and pro-inflammatory than subcutaneous adipose tissue and is more strongly associated with insulin resistance and cardiometabolic complications [87,89]. In contrast, subcutaneous adipose tissue is considered relatively metabolically protective and is associated with improved insulin sensitivity [90]. Importantly, several studies have reported that PAI-1 expression and secretion are higher in visceral adipose depots than in subcutaneous adipose tissue, suggesting that depot-specific differences in adipose biology contribute to variations in protease regulation and cardiometabolic risk [88,91]. Several mechanisms may account for the more pro-inflammatory and metabolically detrimental phenotype of visceral adipose tissue (VAT) compared with subcutaneous adipose tissue (SAT). VAT adipocytes are more lipolytically active and exhibit greater sensitivity to catecholamine-induced lipolysis, resulting in increased release of free fatty acids into the portal circulation and promoting hepatic lipid accumulation, gluconeogenesis, and insulin resistance [92]. In addition, VAT displays greater immune cell infiltration, increased production of pro-inflammatory cytokines such as TNF-α and IL-6, and enhanced hypoxia-associated inflammation [93]. These factors impair insulin signaling through activation of stress kinases, including JNK and IKK/NF-κB, leading to inhibitory serine phosphorylation of IRS-1. Depot-specific differences in extracellular matrix remodeling and vascularization further contribute to these metabolic disparities, as VAT undergoes more extensive fibrosis, matrix stiffening, and hypoxia during obesity, limiting adipocyte expandability and metabolic flexibility [94]. In contrast, SAT exhibits greater adipogenic and angiogenic capacity, facilitating safer lipid storage and reducing ectopic lipid accumulation in non-adipose tissues.

### 4.2. Brown and Beige Adipose Tissue

In contrast to white adipose tissue, brown adipose tissue (BAT) is specialized for thermogenesis and energy expenditure through the oxidation of fatty acids and glucose, a process primarily mediated by the mitochondrial uncoupling protein-1 (UCP1) [95,96]. BAT contains abundant mitochondria and is highly vascularized, features that facilitate efficient heat production and contribute to whole-body energy homeostasis [95,97]. In addition to classical brown adipocytes, inducible thermogenic adipocytes known as beige or “brite” adipocytes can emerge within white adipose depots in response to stimuli such as cold exposure, β-adrenergic signaling, and certain hormonal or metabolic cues [95].

Recent studies indicate that thermogenic adipose tissues also function as metabolically active endocrine organs capable of secreting signaling molecules that influence systemic metabolism and inter-organ communication [98]. Compared with white adipose tissue, brown and beige adipose depots generally display lower inflammatory activity and are associated with improved insulin sensitivity and metabolic health [99,100]. Although the role of serpins in thermogenic adipose tissues has been less extensively characterized than in white adipose tissue, emerging evidence suggests that protease regulation and extracellular matrix remodeling contribute to adipose tissue plasticity during thermogenic activation [101,102].

### 4.3. Regulation of Serpin Expression in Adipose Tissue

The expression of serpins in adipose tissue is regulated by multiple physiological and pathological stimuli, including hypoxia, inflammatory signaling, and metabolic stress [11]. These regulatory mechanisms become particularly relevant during obesity, a condition characterized by extensive structural and functional remodeling of adipose tissue depots [10,11]. As adipose depots expand during excessive nutrient intake, local oxygen availability may become limited, leading to the development of adipose tissue hypoxia [103].

Under normoxic conditions, hypoxia-inducible factor-1α (HIF-1α) is hydroxylated by prolyl hydroxylase domain (PHD) enzymes and targeted for ubiquitination and proteasomal degradation through the von Hippel–Lindau (VHL) E3 ubiquitin ligase complex [11]. Hypoxia suppresses PHD activity, allowing HIF-1α stabilization, nuclear translocation, heterodimerization with HIF-1β, and transcriptional activation of genes involved in angiogenesis, glycolysis, inflammation, and extracellular matrix remodeling. Emerging evidence suggests that Serpina3c may indirectly modulate hypoxia-associated responses through inflammatory and metabolic pathways. In murine adipocytes, Serpina3c suppresses the HIF-1α-glycolysis axis and inhibits Wnt/β-catenin signaling, thereby promoting adipocyte differentiation and attenuating obesity-associated adipose dysfunction [57]. These findings suggest that Serpina3c may act upstream of hypoxia-driven adipose remodeling, although the underlying mechanisms remain incompletely understood [68].

Hypoxic conditions activate hypoxia-inducible factors (HIFs), which regulate the transcription of genes involved in angiogenesis, inflammation, and extracellular matrix remodeling [11]. In addition, emerging evidence suggests that specific adipocyte-expressed serpins, such as Serpina3c, interact with hypoxia-related signaling pathways to influence adipose tissue inflammation and metabolic regulation [68]. Several studies have also shown that hypoxia can increase the expression of PAI-1 in adipocytes, linking oxygen deprivation to enhanced protease inhibition and altered extracellular matrix dynamics within adipose tissue [104,105].

Inflammatory signaling pathways also contribute significantly to the regulation of serpin expression in adipose depots [68]. Obesity is commonly associated with chronic low-grade inflammation characterized by increased infiltration of immune cells, particularly macrophages, into adipose tissue [11]. These immune cells release pro-inflammatory cytokines such as tumor necrosis factor-α (TNF-α) and interleukin-6 (IL-6), which have been shown to stimulate the expression of several serpins, including PAI-1 [106]. In addition to macrophages, adipocytes themselves respond to inflammatory stimuli by increasing serpin production, indicating that both immune-derived and adipocyte-derived signals contribute to the regulation of protease inhibitors within adipose tissue [106].

Metabolic stress associated with obesity and insulin resistance represents another important factor influencing serpin expression [11]. Elevated levels of circulating free fatty acids, hyperglycemia, and oxidative stress can activate intracellular signaling pathways that alter gene transcription in adipocytes [11]. These metabolic signals have been shown to increase PAI-1 expression in adipose tissue, linking metabolic dysfunction with alterations in proteolytic balance and extracellular matrix turnover [107]. Collectively, these regulatory mechanisms highlight the complex interplay between hypoxic, inflammatory, and metabolic signals that control serpin expression in adipose tissue and contribute to adipose tissue remodeling during metabolic disease [11,108].

### 4.4. Lean vs. Obese and Insulin-Resistant States

Adipose tissue undergoes substantial functional and molecular alterations during the transition from lean to obese states, which can significantly influence the expression of several members of the serpin family [109,110]. In lean individuals, adipose tissue typically exhibits relatively low levels of inflammatory signaling and balanced extracellular matrix turnover, conditions that support normal metabolic homeostasis and insulin sensitivity [111,112]. Under these physiological conditions, serpin expression within adipose depots remains tightly regulated, contributing to the maintenance of proteolytic balance and controlled tissue remodeling [109,110].

In contrast, obesity is characterized by adipose tissue expansion, increased immune cell infiltration, and chronic low-grade inflammation, all of which contribute to altered serpin expression profiles [10,113]. Several studies have reported that the expression of plasminogen activator inhibitor-1 (PAI-1), one of the most extensively studied adipose-derived serpins, is significantly elevated in obese and insulin-resistant individuals [10,114]. Elevated PAI-1 levels are thought to contribute to impaired fibrinolysis, enhanced extracellular matrix deposition, and progressive adipose tissue fibrosis, processes that may further exacerbate metabolic dysfunction [114].

Furthermore, increased serpin expression in obesity has been linked to systemic metabolic disturbances, including insulin resistance, dyslipidemia, and cardiovascular complications [53]. Experimental studies have demonstrated that elevated PAI-1 levels in adipose tissue correlate with markers of metabolic syndrome and may contribute to the pathogenesis of obesity-related cardiometabolic disease [85,115]. Together, these findings indicate that dysregulated serpin expression represents an important molecular feature distinguishing metabolically healthy adipose tissue from the inflamed and dysfunctional adipose tissue observed in obesity and insulin-resistant states, highlighting the role of protease regulation in adipose tissue remodeling and cardiometabolic disease [53].

## 5. Serpins in Extracellular Matrix Remodeling and Fibrotic Transition

Here, we focus on serpin biology in adipose tissue, prioritizing evidence from adipose depots while distinguishing human, rodent, and non-adipose findings. Mechanistic insights from other tissues are included only when relevant to adipose ECM remodeling, fibrosis, or metabolic dysfunction [116]. In particular, the role of serpins, particularly PAI-1, in adipose extracellular matrix remodeling and metabolic dysfunction is highlighted.

Extracellular matrix (ECM) remodeling is a fundamental process that regulates adipose tissue expansion, structural integrity, and metabolic function [117]. During physiological tissue growth, ECM turnover is tightly controlled through the coordinated activity of proteases and their endogenous inhibitors [118]. Among these regulatory molecules, members of the serpin superfamily play a critical role in modulating proteolytic pathways that govern fibrinolysis, matrix degradation, and tissue remodeling [119]. In adipose tissue, dysregulation of serpin-mediated protease inhibition has increasingly been recognized as an important contributor to excessive extracellular matrix accumulation, tissue fibrosis, and impaired metabolic homeostasis [117]. In particular, PAI-1, one of the most extensively studied serpins in metabolic tissues, acts as a central regulator of plasmin generation and downstream matrix remodeling processes [120]. Elevated expression of serpins during obesity and metabolic stress disrupts the balance between ECM synthesis and degradation, thereby promoting fibrotic remodeling and adipose tissue dysfunction [118].

### 5.1. PAI-1 and Plasmin Inhibition

PAI-1 is the primary physiological inhibitor of tissue-type and urokinase-type plasminogen activators (tPA and uPA), which convert plasminogen into the active protease plasmin [121]. Plasmin plays a pivotal role in extracellular matrix turnover by directly degrading fibrin and by activating several matrix metalloproteinases (MMPs), which further contribute to collagen degradation and matrix remodeling [122]. Through inhibition of tPA and uPA, PAI-1 limits plasmin generation and consequently reduces proteolytic ECM degradation [117].

Within adipose tissue, increased PAI-1 expression disrupts the balance between proteolysis and matrix deposition [85]. Elevated PAI-1 levels reduce plasmin activity, thereby decreasing activation of matrix-degrading enzymes such as MMPs [123]. This reduction in proteolytic activity promotes the accumulation of fibrin and other extracellular matrix components within adipose depots [124]. As a result, excessive PAI-1 expression shifts the balance toward matrix stabilization and deposition rather than degradation [117].

Experimental studies have demonstrated that PAI-1 expression is markedly increased in adipose tissue during obesity and metabolic stress [85]. Both adipocytes and stromal vascular cells contribute to the production of PAI-1 within adipose depots [125]. Increased PAI-1 activity not only inhibits fibrinolysis but also promotes structural changes in the adipose tissue microenvironment, including increased matrix rigidity and impaired tissue remodeling [117]. These alterations may restrict healthy adipose tissue expansion and contribute to fibrotic remodeling of adipose depots [117].

### 5.2. Collagen Deposition and Tissue Stiffness

In adipose tissue, as demonstrated in human and mouse studies, fibrotic remodeling is characterized by excessive deposition of ECM proteins, particularly fibrillar collagens such as collagen types I, III, and VI [126,127]. In addition to fibrillar collagens, other ECM components such as fibronectin and lysyl oxidase (LOX)-mediated crosslinking enzymes contribute to adipose tissue fibrotic remodeling [128,129]. Fibronectin functions as an important scaffold protein that facilitates collagen assembly and matrix organization, while LOX enzymes catalyze collagen crosslinking that stabilizes the ECM and increases tissue stiffness [128,130]. Increased expression of fibronectin and LOX has been observed in obese adipose tissue and is associated with enhanced matrix rigidity and impaired adipose tissue expandability [129,130,131]. These alterations further reinforce the fibrotic microenvironment and contribute to the mechanical constraints that limit healthy adipose tissue remodeling [128,130,131]. While these mechanisms have been extensively characterized in multiple fibrotic tissues, similar processes have been reported in adipose tissue [4].

Under physiological conditions, ECM turnover maintains tissue flexibility and allows adipocytes to expand in response to energy storage demands. However, dysregulation of protease activity resulting from increased serpin expression disrupts this balance and promotes progressive collagen accumulation. Within adipose tissue, serpins such as PAI-1 contribute to this imbalance by limiting protease-mediated ECM degradation [5,6].

Elevated PAI-1 levels inhibit plasmin-mediated activation of matrix metalloproteinases responsible for degrading collagen fibers and other ECM components [132]. Reduced MMP activity consequently limits collagen degradation, leading to excessive matrix deposition within adipose tissue [133]. Over time, this process results in increased tissue stiffness and reduced structural plasticity [133].

Accumulation of collagen fibers alters the mechanical properties of adipose tissue by increasing extracellular matrix rigidity [126]. This stiffened ECM environment constrains adipocyte hypertrophy and reduces the expandability of adipose tissue depots [134]. Limited adipose expandability has been proposed as a key mechanism linking adipose fibrosis to metabolic dysfunction [134,135]. When adipocytes are unable to expand appropriately, excess lipids may accumulate in ectopic tissues such as the liver and skeletal muscle, thereby contributing to systemic metabolic disturbances [136].

Furthermore, increased ECM stiffness influences cellular signaling pathways within adipose tissue [126]. Mechanical stress generated by a rigid extracellular matrix can activate mechanosensitive signaling pathways that alter adipocyte differentiation, inflammatory responses, and metabolic function [126].

### 5.3. Fibrosis as a Driver of Metabolic Dysfunction

Adipose tissue fibrosis has emerged as a critical pathological feature linking obesity to metabolic disease [137,138]. Fibrotic remodeling limits the capacity of adipose tissue to safely store excess lipids, leading to adipocyte dysfunction and ectopic lipid deposition in non-adipose organs, particularly the liver, thereby contributing to the development of metabolic complications such as non-alcoholic fatty liver disease (NAFLD) [139]. In this context, serpins such as PAI-1 contribute to fibrosis by suppressing protease activity and promoting extracellular matrix accumulation [136].

Fibrosis within adipose tissue is frequently accompanied by increased infiltration of immune cells, including macrophages and other inflammatory cell populations [118,140]. These immune cells release cytokines and profibrotic mediators that stimulate fibroblast activation and collagen synthesis [118,141,142]. In parallel, elevated serpin expression further reduces ECM degradation, thereby amplifying fibrotic remodeling processes [136].

The presence of fibrosis alters adipocyte metabolic function through several mechanisms [136]. Excess ECM deposition restricts adipocyte expansion, resulting in increased cellular stress, hypoxia, and inflammatory signaling within adipose tissue [136,143]. These pathological changes impair insulin signaling pathways and contribute to the development of systemic insulin resistance [144,145]. In addition, fibrotic adipose tissue exhibits reduced adipogenic capacity, limiting the formation of new adipocytes that could otherwise accommodate excess lipid storage [136,145].

Clinical and experimental studies have demonstrated that increased adipose tissue fibrosis correlates with insulin resistance, dyslipidemia, and other features of metabolic syndrome [146,147]. Therefore, fibrosis is increasingly viewed not merely as a consequence of obesity but also as an active contributor to metabolic dysfunction [138,146].

### 5.4. Mechanotransduction and Insulin Signaling Interference

The mechanical properties of the extracellular matrix play an important role in regulating cellular behavior through a process known as mechanotransduction [148,149]. In fibrotic adipose tissue, increased matrix stiffness alters the mechanical cues transmitted to adipocytes and stromal cells [150]. These mechanical signals are sensed by cell-surface receptors such as integrins, which connect the extracellular matrix to intracellular cytoskeletal structures and signaling pathways [151,152].

Activation of mechanotransduction pathways in response to increased matrix rigidity can profoundly affect adipocyte function [153,154]. Many of these pathways have been characterized in other cell types and tissues and are inferred to operate similarly in adipose tissue [153]. Integrin-mediated signaling activates downstream pathways including focal adhesion kinase (FAK), Rho-associated kinase (ROCK), and other cytoskeletal regulators that influence cellular metabolism and differentiation [152,153]. Excessive activation of these pathways has been shown to impair insulin signaling and promote inflammatory responses within adipose tissue [154,155].

Matrix stiffness may interfere with insulin receptor signaling by altering cytoskeletal organization and intracellular signaling cascades, as the mechanical properties of the extracellular matrix regulate cellular behavior through mechanotransduction pathways [156]. Mechanical stress generated by a rigid ECM can disrupt insulin receptor substrate (IRS) signaling and downstream pathways such as phosphatidylinositol-3-kinase (PI3K) and Akt, which are essential for glucose uptake and metabolic regulation [157,158]. Activation of mechanosensitive signaling pathways, including focal adhesion kinase (FAK), RhoA/ROCK, and associated cytoskeletal regulators, has been shown to interfere with insulin signaling and metabolic responses [155,158]. Consequently, the fibrotic extracellular matrix environment contributes to the development of insulin resistance in adipocytes by promoting signaling alterations that impair insulin action [156,157].

Mechanotransduction signaling also influences adipocyte differentiation and lipid metabolism. Increased matrix stiffness has been associated with reduced adipogenic differentiation and altered expression of metabolic genes, thereby contributing to adipose tissue dysfunction [159]. Stiff ECM environments modulate cytoskeletal dynamics and transcriptional regulators such as Yes-associated protein (YAP) and transcriptional co-activator with PDZ-binding motif (TAZ), which integrate mechanical cues to regulate cell fate decisions including differentiation and metabolism [160,161]. These mechanisms remain partially speculative in adipose tissue and require further direct experimental validation [155]. Overall, these findings highlight how matrix stiffness-driven mechanotransduction pathways disrupt insulin signaling and adipocyte function, thereby promoting metabolic dysregulation linked to insulin resistance [157,160,161]. Collectively, these findings demonstrate that serpin-mediated regulation of proteolytic pathways plays a central role in controlling extracellular matrix remodeling and adipose tissue fibrosis [136]. Through effects on matrix turnover, mechanical signaling, and insulin signaling pathways, dysregulated serpin expression contributes to the structural and metabolic alterations that characterize obesity-associated adipose tissue dysfunction [119,136].

## 6. Serpins as Modulators of Adipose Immune Cell Dynamics

Adipose tissue is increasingly recognized as an immunologically active organ in which immune cells and adipocytes interact to regulate metabolic homeostasis and inflammatory responses [162]. In addition to structural remodeling of the extracellular matrix described in the previous section, obesity-induced adipose tissue dysfunction is strongly associated with profound alterations in immune cell composition and inflammatory signaling [10]. In obesity, expansion of adipose depots is accompanied by extensive immune cell infiltration, particularly macrophages, which contribute to the establishment of chronic low-grade inflammation known as metaflammation [10]. This inflammatory environment alters adipose tissue structure and metabolic function, thereby promoting insulin resistance and systemic metabolic dysfunction [10,68]. Members of the serpin superfamily have emerged as important regulators of these processes through their ability to modulate protease activity, inflammatory signaling pathways, and immune cell behavior within adipose tissue [10,163]. Several serpins, including plasminogen activator inhibitor-1 (PAI-1; *SERPINE1*), SerpinA1, SerpinA3, and (PEDF; *SERPINF1*), have been implicated in the regulation of immune cell recruitment, macrophage polarization, and cytokine production in metabolic tissues [10,163,164]. Through their capacity to influence both proteolytic signaling and inflammatory networks, serpins contribute to the dynamic interplay between adipocytes and immune cells that characterize obese adipose tissue [10,163].

### 6.1. Macrophage Recruitment and Retention

**Figure 3:**
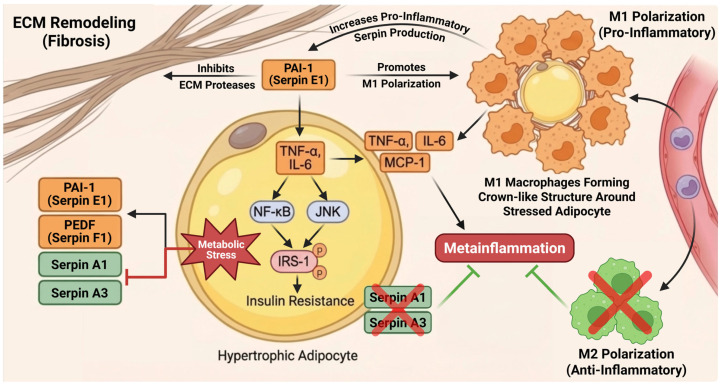
Serpins orchestrate immune–metabolic crosstalk in adipose tissue. During obesity, adipose tissue expansion induces metabolic stress, increasing the expression of pro-inflammatory serpins, including plasminogen activator inhibitor-1 (PAI-1; SERPINE1) and pigment epithelium-derived factor (PEDF; SERPINF1), while protective serpins such as SERPINA1 and SERPINA3 decline. Elevated PAI-1 inhibits extracellular matrix (ECM) proteases, promoting fibrosis and inflammatory signaling that contribute to IRS-1 phosphorylation and insulin resistance. Stressed adipocytes release inflammatory mediators that, together with PAI-1, promote monocyte recruitment and macrophage activation, favoring polarization toward a pro-inflammatory M1 phenotype characterized by increased tumor necrosis factor-α (TNF-α), interleukin-6 (IL-6), and monocyte chemoattractant protein-1 (MCP-1). M1 macrophages accumulate around stressed adipocytes, forming crown-like structures and further enhancing pro-inflammatory serpin production. Together, these interactions amplify adipose inflammation and contribute to the chronic low-grade inflammatory state known as metaflammation. Image created with https://www.biorender.com accessed on 8 April 2026.

Macrophages represent one of the most abundant immune cell populations in obese adipose tissue and play a central role in metabolic inflammation [165,166,167]. During obesity, circulating monocytes are recruited to expanding adipose depots where they differentiate into adipose tissue macrophages and accumulate around stressed or dying adipocytes, forming characteristic crown-like structures [166,167,168]. The recruitment and retention of macrophages are mediated by chemokines, extracellular matrix remodeling, and protease-dependent signaling pathways that regulate cell migration within the adipose microenvironment (Figure 3) [74,168].

Serpins can influence macrophage recruitment by regulating proteolytic pathways that control cell migration and extracellular matrix degradation. Evidence for these mechanisms has been demonstrated in adipose tissue, primarily in rodent models, with supporting observations from human adipose tissue studies [116]. PAI-1, in particular, has been shown to modulate leukocyte trafficking through its ability to inhibit plasmin generation and alter extracellular matrix turnover. Elevated PAI-1 levels in obese adipose tissue have been associated with increased macrophage accumulation and enhanced inflammatory signaling [164,169]. In addition to PAI-1, other serpins such as SerpinA1 and SerpinA3 have been reported, primarily in non-adipose inflammatory systems and metabolic tissues, to regulate inflammatory responses and immune cell recruitment through modulation of protease activity and cytokine signaling [170,171].

Furthermore, protease inhibition by serpins can affect macrophage retention within adipose tissue by modifying the structural properties of the extracellular matrix [10]. Increased extracellular matrix deposition and reduced proteolytic activity promote the formation of a fibrotic microenvironment that supports immune cell persistence within adipose depots [172]. As a result, elevated serpin expression may contribute to sustained macrophage accumulation and prolonged inflammatory signaling in obese adipose tissue [10]. In addition to PAI-1, pigment epithelium-derived factor (PEDF; SERPINF1) has emerged as an important mediator of obesity-associated metabolic dysfunction. Elevated circulating PEDF levels have been associated with obesity, insulin resistance, and type 2 diabetes, where PEDF promotes lipolysis, free fatty acid release, and inflammatory responses. Mechanistically, PEDF activates JNK and NF-κB pathways, enhances inhibitory serine phosphorylation of IRS-1, and impairs insulin signaling [173]. PEDF has also been linked to macrophage activation and adipose tissue inflammation, supporting a role for SERPINF1 in the interplay between local adipose dysfunction and systemic metabolic disturbances [81].

### 6.2. M1/M2 Polarization Balance

Macrophages within adipose tissue can adopt distinct functional phenotypes depending on environmental signals, commonly classified as pro-inflammatory M1 macrophages and anti-inflammatory M2 macrophages. Lean adipose tissue is typically enriched in M2-like macrophages that support tissue remodeling, anti-inflammatory signaling, and metabolic homeostasis [174]. In contrast, obesity promotes a shift toward M1-like macrophage polarization, characterized by increased production of pro-inflammatory cytokines such as tumor necrosis factor-α (TNF-α), interleukin-6 (IL-6), and interleukin-1β (IL-1β) [175,176].

Macrophage polarization dynamics are well established in human and mouse adipose tissue [4] and are influenced by multiple microenvironmental factors, including hypoxia, adipocyte-derived cytokines, and metabolic stress signals that modulate macrophage phenotype and contribute to insulin resistance [176,177]. In addition, emerging evidence indicates that signaling pathways such as neuropeptide Y1 receptor signaling and microRNA-mediated regulation can influence macrophage polarization and inflammatory responses in adipose tissue [178,179]. Targeting these pathways has shown potential to attenuate chronic inflammation associated with obesity [178,179]. Overall, the dynamic balance between M1 and M2 macrophages represents a key regulatory mechanism governing adipose tissue inflammation and metabolic homeostasis during obesity [177].

Emerging evidence suggests that serpins can influence macrophage polarization through their effects on inflammatory signaling pathways and protease activity [180,181,182]. However, much of this evidence is derived from non-adipose immunological and metabolic systems and may not fully reflect adipose-specific mechanisms [7]. PAI-1 has been implicated in promoting pro-inflammatory macrophage activation and may contribute to the predominance of M1 macrophages in obese adipose tissue. Increased PAI-1 expression has been associated with elevated cytokine production and enhanced inflammatory responses within metabolic tissues [164,169]. Through modulation of proteolytic signaling and inflammatory cascades, serpins may therefore influence shifts between pro-inflammatory and anti-inflammatory macrophage states within adipose tissue, contributing to the regulation of local immune responses during obesity [180,181].

Other serpins may exert protective or regulatory roles in macrophage polarization. SerpinA1, also known as α1-antitrypsin, possesses anti-inflammatory properties and has been shown to suppress excessive immune activation and cytokine release [183,184]. Similarly, SerpinA3 has been implicated, primarily in non-adipose inflammatory and injury models, in modulating inflammatory signaling pathways and regulating macrophage responses during tissue injury and metabolic stress [68]. Through these mechanisms, serpins may influence the balance between pro- and anti-inflammatory macrophage phenotypes within adipose tissue [68].

### 6.3. Effects on Cytokine Networks

Cytokine signaling networks play a critical role in coordinating immune responses within adipose tissue and mediating communication between immune cells and metabolic tissues [167]. In obesity, adipose tissue becomes a major source of pro-inflammatory cytokines, including TNF-α, IL-6, monocyte chemoattractant protein-1 (MCP-1), and other inflammatory mediators that contribute to systemic metabolic dysfunction [141,167].

Serpins can regulate these cytokine networks by modulating protease-dependent signaling pathways and inflammatory cascades [185]. PAI-1 has been shown to amplify inflammatory signaling by promoting the production of cytokines and chemokines that recruit additional immune cells into adipose tissue [85]. Increased PAI-1 expression correlates with elevated levels of inflammatory mediators and has been linked to the development of insulin resistance and metabolic syndrome [85,186].

These cytokine alterations are well documented in human and mouse adipose tissue during obesity [187,188]. In contrast, certain serpins exhibit anti-inflammatory activities that may counterbalance excessive cytokine production. SERPINA1 has been reported to inhibit inflammatory cytokine release and reduce tissue inflammation through its protease-inhibitory and immunomodulatory functions [189]. Most of these findings originate from broader immunological and non-adipose tissue studies [188]. Pigment epithelium-derived factor (SERPINF1/PEDF) also participates in inflammatory signaling networks and has been implicated in the regulation of macrophage activation and cytokine production in metabolic tissues [7,190]. Through these diverse effects, serpins contribute to the regulation of cytokine networks that shape the inflammatory environment of adipose tissue [191].

### 6.4. Crosstalk Between Adipocytes and Immune Cells

The interaction between adipocytes and immune cells represents a central feature of adipose tissue immunometabolism [192]. Adipocytes not only serve as energy-storing cells but also act as endocrine and immunomodulatory cells that secrete adipokines, cytokines, and protease regulators that influence immune cell function [193]. In obesity, stressed adipocytes release inflammatory mediators and danger signals that promote immune cell recruitment and activation [194].

Serpins participate in this bidirectional communication by regulating proteolytic signaling pathways that influence both adipocyte and immune cell function [10]. PAI-1 produced by adipocytes can influence macrophage migration, inflammatory signaling, and extracellular matrix remodeling within adipose depots [10]. In turn, macrophage-derived cytokines can stimulate adipocytes to increase serpin production, establishing a feedback loop that amplifies inflammatory responses (Figure 3) [10].

Additional serpins also contribute to adipocyte–immune cell communication. SerpinA1 and SerpinA3 may modulate, based largely on evidence from non-adipose and inflammatory systems, signaling and protease activity within the adipose microenvironment, thereby influencing immune cell activation and tissue remodeling [10,68]. Meanwhile, SERPINF1/PEDF has been shown to regulate adipocyte metabolism and macrophage behavior through mechanisms involving inflammatory signaling and lipid metabolism [7,190,195]. Together, these interactions highlight the multifaceted roles of serpins in coordinating the complex communication network between adipocytes and immune cells that drive chronic inflammation and metabolic dysfunction in obesity (Figure 3) [10,68,185]. Some of these roles remain partially speculative in adipose tissue and require further direct investigation.

## 7. Angiogenesis, Hypoxia, and Vascular Remodeling

Angiogenesis is needed to maintain healthy adipose tissue growth since the growth of the fat depots needs a sufficient blood stream to supply oxygen and nutrients and to eliminate metabolic waste [3,9]. In metabolically healthy adipose tissue, vascular remodeling accompanies tissue expansion and supports adipocyte function and endocrine signaling. In obesity, this process becomes insufficient, and adipose expansion frequently outpaces vascular growth that leads to reduced perfusion and local hypoxia [113]. Angiogenesis therefore functions not merely as a structural process but as a critical determinant of adipose metabolic homeostasis [3].

Hypoxia represents a principal consequence of inadequate vascular adaptation. In obesity, expanding adipose tissue frequently exceeds its blood supply, resulting in areas of decreased oxygen tension within the tissue. When hypoxia occurs, hypoxia-inducible factor-1 alpha or HIF-1α is activated, and it alters the expression of inflammatory, fibrotic, and metabolic regulatory genes [3]. Notably, it has been demonstrated that this hypoxic response may not generate an adequate proangiogenic repair in adipose tissue. In fact, it tends to induce fibrotic remodeling and inflammatory stimulation, deteriorating adipose dysfunction and not homeostasis [3].

Hypoxia contributes to metabolic dysfunction by impairing insulin signaling in adipocytes. Low oxygen levels in adipocytes decrease phosphorylation of the insulin receptor and downstream signaling transducers including protein kinase B, and produce an insulin resistance state [196]. The significance of this observation lies in the fact that hypoxia is not merely a condition that is correlated with insulin resistance, proving to be capable of acting as a cause of cellular insulin defects. In line with this, Lee et al. (2014) [4] revealed that the enhancement of adipocyte oxygen consumption in obesity itself could induce local hypoxia, HIF-1α stimulation, the release of chemokines, adipose inflammation, and resistance to insulin in the body.

Within this vascular insufficiency and hypoxia context, serpins emerge as regulators of angiogenic balance in adipose tissue. PEDF is a notable example of an anti-angiogenic serpin, and its elevated levels in obesity suggest a role in the broader failure of vascular adaptation during adipose expansion [7]. Simultaneously, PEDF has been indicated to enhance insulin resistance and inflammatory signaling, suggesting that PEDF is implicated not only in impaired angiogenic regulation but also in broader immune–metabolic dysfunction [6].

Pigment epithelium-derived factor *SERPINF1*-coded PEDF is a powerful anti-angiogenic serpin, which is involved in the regulation of adipose tissue vascular development. PEDF prevents angiogenesis in a mechanism involving inhibition of endothelial cell proliferation, migration, and survival, thus restricting new blood vessel growth. Increased levels of PEDF have also been observed in adipose tissue and the circulation of obese individuals, and it has been found that this serpin plays a role in vascular maladaptation during adipose growth [6,7]. Impaired angiogenesis may limit oxygen delivery, thereby promoting local hypoxia and initiating downstream fibrotic and inflammatory responses. Moreover, elevated PAI-1 promotes ECM accumulation and reduces proteolytic turnover, creating a fibrotic and mechanically restrictive microenvironment that impairs endothelial cell migration and vessel remodeling. PAI-1 is also induced under hypoxic conditions and participates in inflammatory signaling that links vascular dysfunction to immune activation. Together these serpins show that vascular maladaptation in adipose tissue arises not only from insufficient pro-angiogenic signaling but from an imbalance between anti-angiogenic activity (PEDF) and ECM-driven structural constraints (PAI-1).

## 8. Molecular Links to Insulin Sensitivity

Insulin sensitivity reflects the ability of cells to respond effectively to insulin and is a central determinant of metabolic health. Its impairment, termed insulin resistance, is a hallmark of obesity, type 2 diabetes, and metabolic syndrome. Insulin resistance arises through interconnected mechanisms involving chronic inflammation, extracellular matrix remodeling, adipokine imbalance, lipotoxicity, and altered immune signaling. Within this network, members of the serpin superfamily have emerged as important modulators of adipose tissue homeostasis. Current evidence suggests that serpins influence insulin sensitivity predominantly through indirect mechanisms, including regulation of inflammatory signaling, fibrosis, angiogenesis, and lipid metabolism, rather than through direct interaction with canonical insulin-signaling components. Accordingly, this section examines how serpin-mediated alterations in the adipose tissue microenvironment contribute to impaired insulin responsiveness and systemic metabolic dysfunction.

### 8.1. Inflammatory Interference with Insulin Signaling

Chronic, low-grade inflammation is a hallmark of obesity and a primary driver of insulin resistance. This inflammatory state is characterized by the overproduction of pro-inflammatory cytokines within adipose tissue, which directly interfere with the intracellular signaling cascade of insulin [197]. IRS-1 serine phosphorylation is a key mechanism by which inflammation disrupts insulin’s action. Normally, upon insulin stimulation, IRS proteins, particularly IRS-1, become phosphorylated on tyrosine residues, allowing them to transmit the signal downstream. However, pro-inflammatory stimuli activate specific serine/threonine kinases that instead phosphorylate IRS-1 on serine residues. This serine phosphorylation acts as a negative regulator, preventing the necessary tyrosine phosphorylation and thereby uncoupling the insulin receptor from its downstream signaling effectors, leading to insulin resistance [197]. Two major inflammatory pathways involved in this process are the NF-κB and JNK pathways. Importantly, serpins such as PEDF (SERPINF1) play a critical role in activating these inflammatory pathways. Studies have shown that PEDF (SerpinF1) in adipocytes, skeletal muscle cells, and cultured myotubes is associated with the activation of c-Jun N-terminal kinase (JNK) and IκB kinase (IKK), which in turn activates NF-κB. JNK is an inhibitory Ser/Thr kinase that directly phosphorylates IRS-1 on serine/threonine residues [198], while IKK plays a central role in insulin resistance by forming complexes with IRS-1, contributing to TNFα-mediated insulin resistance [199]. Taken together, these findings demonstrate how serpins such as PEDF contribute to insulin resistance by activating inflammatory pathways, which promote the serine phosphorylation of IRS-1 that disrupts insulin signaling.

### 8.2. ECM Stiffness and Insulin Resistance

The ECM is not merely a structural scaffold but an active participant in cellular signaling, and its composition can influence insulin sensitivity. Serpins are central regulators of ECM remodeling, linking tissue architecture to metabolic signaling. A key player in this process is PAI-1, a serpin that inhibits the plasminogen activators tPA and uPA, thereby reducing fibrinolysis and influencing ECM remodeling [49].

PAI-1 (SERPINE1) is synthesized by adipocytes, particularly in human visceral adipose tissue, and its levels are elevated in obese individuals and associated with insulin resistance [200]. The activity of PAI-1 is stabilized by binding to the ECM protein vitronectin (VN), which protects it from inhibition and prevents its spontaneous conversion to a latent, inactive form, making this interaction crucial for PAI-1’s function and stability within the ECM [201]. Notably, elevated PAI-1 levels in obesity inhibit plasminogen activators, leading to reduced plasmin production and decreased degradation of ECM components [201]. This can contribute to ECM stiffness within adipose tissue, which is known to promote insulin resistance [136]. Experimental silencing of PAI-1 has been shown to prevent TNFα-induced inhibition of adipocyte glucose uptake, supporting a direct role for PAI-1 in adipocyte insulin resistance beyond extracellular matrix remodeling [202]. Elevated PAI-1 and TNFα levels have also been associated with reduced PPARγ expression, thereby suppressing adipogenic and insulin-sensitizing pathways [202]. In addition, PAI-1 may participate in a pro-inflammatory autocrine loop that further amplifies TNFα production within adipose tissue, contributing to persistent metaflammation and metabolic dysfunction [202]. Although pharmacological inhibition of PAI-1 represents a promising therapeutic strategy, prolonged suppression of PAI-1 activity may require caution because excessive adipose lipid mobilization could potentially promote lipotoxicity in non-adipose organs [203]. Furthermore, PAI-1 is itself a potent regulator of hepatic lipid metabolism. RNA sequencing revealed that pharmacological inhibition or genetic reduction of PAI-1 directly regulates the transcriptional expression of numerous genes involved in lipid homeostasis, including downregulating Pcsk9 and upregulating Fgf21 [204]. Recent clinical studies support these findings, showing that circulating PAI-1 levels are positively correlated with liver steatosis and fibrosis in overweight and obese individuals independent of age, gender, and insulin resistance [205].

In summary, elevated levels of adipocyte-derived PAI-1 (SERPINE1) are associated with obesity, insulin resistance, and hepatic steatosis. By reducing fibrinolysis, disrupting ECM dynamics, and directly regulating hepatic lipid metabolism, increased PAI-1 levels are associated with a pro-fibrotic and lipotoxic microenvironment that may contribute to the persistence of insulin resistance.

### 8.3. Adipokine Modulation

Adipose tissue functions as a major endocrine organ, secreting adipokines that can either enhance or impair insulin sensitivity. Several non-serpin adipokines, including chemerin, visfatin, and omentin-1, are also involved in obesity-associated metabolic dysfunction [206]. Chemerin and visfatin are generally linked to pro-inflammatory and insulin-resistant states, whereas omentin-1 is considered predominantly anti-inflammatory and insulin-sensitizing [206]. Within this broader adipokine network, adipose-related serpins such as vaspin (SERPINA12), kallistatin (SERPINA4), and PEDF (SERPINF1) have emerged as important modulators of metabolic inflammation, adipose tissue function, and insulin sensitivity.

Vaspin (SERPINA12) is a visceral adipose tissue-derived serine protease inhibitor that possesses insulin-sensitizing effects [69]. Its expression in human adipose tissue is induced by obesity and may represent a compensatory mechanism associated with insulin resistance [70]. Administration of recombinant vaspin has been shown to improve insulin sensitivity and glucose tolerance in diet-induced obese mice [71]. The beneficial effect of vaspin on glucose metabolism is at least in part mediated by its serpin activity, as non-inhibitory vaspin mutants fail to improve glucose tolerance [72]. One mechanism involves vaspin’s inhibition of the protease kallikrein 7 (KLK7), which can degrade insulin, thereby extending insulin half-life and activity [73]. KLK7 also propagates infiltration and activation of inflammatory macrophages in visceral adipose tissue, suggesting that vaspin’s inhibition of KLK7 may contribute to reducing adipose tissue inflammation [74]. Notably, vaspin can be internalized via the LRP1 receptor and bind DNA, further enhancing KLK7 inhibition, representing a newly discovered mechanism of action [75]. Moreover, vaspin also exerts its effects by binding to the cell-surface receptor GRP78, thereby attenuating endoplasmic reticulum stress in obesity, a known contributor to insulin resistance [76]. These multi-faceted effects highlight how a single serpin can coordinate protease inhibition, inflammatory control, and cellular stress responses, ultimately contributing to metabolic homeostasis. Vaspin also modulates obesity-related metabolic pathways by normalizing leptin, resistin, and TNFα expression while increasing GLUT4 and adiponectin in white adipose tissue [207]. It further protects vascular endothelial cells through PI3K/Akt-dependent mechanisms and inhibits KLK7 through a classical serpin mechanism, which may reduce adipose inflammation and immune-cell infiltration [73,74,208]. Clinically, circulating vaspin levels correlate with obesity and insulin resistance, suggesting a compensatory protective response and supporting vaspin as a potential therapeutic target in obesity-associated metabolic disease [73,207].

Similarly, kallistatin (SERPINA4) has emerged as a protective factor. Circulating kallistatin levels are significantly reduced in obese patients and increase after weight loss achieved by Roux-en-Y gastric bypass [62]. Kallistatin treatment in human adipocytes reduces the expression of inflammatory cytokines and upregulates the expression of anti-inflammatory and insulin-sensitizing genes such as ADIPOQ and KLF4. Mechanistically, kallistatin inhibits TNF-α- and LPS-induced inflammation and reduces oxidative stress by downregulating NOX2 and HIF1a while promoting SIRT1 and FOXO1 expression in adipose tissue [62].

Conversely, PEDF (SERPINF1) plays a causal role in the development of insulin resistance [79]. Elevated circulating PEDF levels are positively associated with metabolic risk factors like BMI, fasting insulin, and HOMA-IR [80]. PEDF acts as a lipolytic factor, promoting free fatty acid (FFA) mobilization from adipose tissue, which can lead to ectopic lipid deposition and inflammation [81]. It also impairs insulin signal transduction by activating the JNK and IKK pathways, leading to serine phosphorylation of IRS-1 [6].

Collectively, the balance of adipokines is critical for systemic insulin sensitivity. Vaspin acts as an insulin sensitizer, in part by inhibiting proteases like KLK7 that degrade insulin and promote inflammation, while PEDF promotes insulin resistance by inducing lipolysis and activating inflammatory pathways. Kallistatin serves as a protective, anti-inflammatory adipokine whose levels are reduced in obesity, highlighting the therapeutic potential of restoring its function.

Current evidence indicates that serpins exert both protective and pathogenic effects in adipose tissue and metabolic disease. Protective serpins, including vaspin (SERPINA12), kallistatin (SERPINA4), and α1-antitrypsin (SERPINA1), generally mitigate inflammation, oxidative stress, and insulin resistance. In contrast, PAI-1 (SERPINE1) and PEDF (SERPINF1) have been associated with extracellular matrix remodeling, fibrosis, inflammatory activation, and impaired insulin sensitivity, whereas SERPINA3 may contribute to chronic inflammatory responses during obesity [49].

Mechanistically, obesity-associated serpin dysregulation promotes adipose tissue dysfunction through complementary pathways. PAI-1 impairs plasmin-dependent matrix turnover, favoring extracellular matrix accumulation and fibrosis, whereas PEDF activates JNK and NF-κB signaling, enhances inhibitory serine phosphorylation of IRS-1, and disrupts insulin signaling while promoting lipolysis and free fatty acid release [49]. Collectively, these processes contribute to chronic adipose inflammation and metabolic impairment.

Overall, adipose-associated serpins participate in interconnected networks regulating ECM remodeling, inflammatory responses, vascular adaptation, and insulin sensitivity, with their effects influenced by adipose depot characteristics and the local metabolic environment [157,204,205,206].

### 8.4. Systemic Effects: Inter-Organ Crosstalk in Insulin Resistance

**Figure 4:**
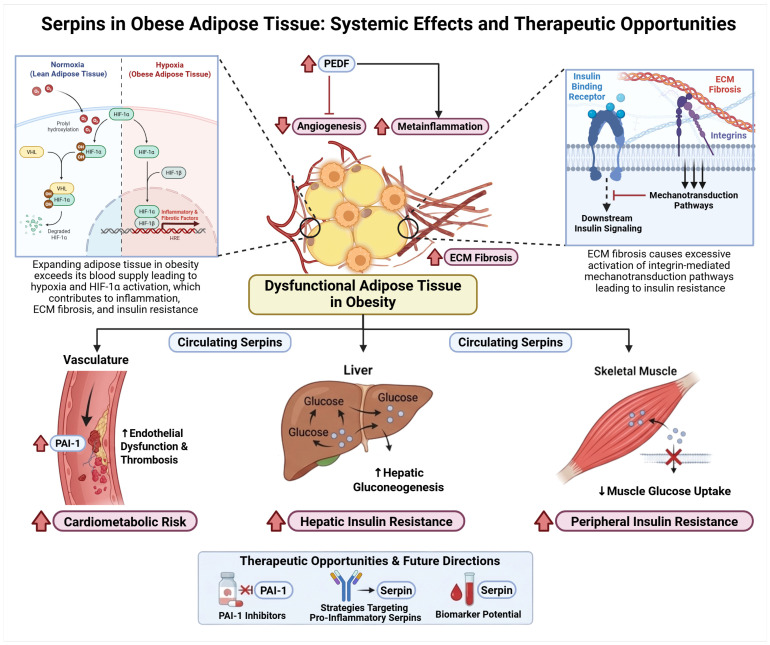
Serpins in Obese Adipose Tissue: Systemic Effects and Therapeutic Opportunities. Obesity drives adipose tissue hypoxia and hypoxia-inducible factor 1α (HIF-1α) activation, promoting inflammation and ECM (ECM) fibrosis. Pigment epithelium-derived factor (PEDF) is an adipocyte-derived anti-angiogenic and pro-inflammatory serpin, which perpetuates adipose tissue hypoxia and metainflammation. Increased ECM stiffness in obesity overactivates integrin-mediated mechanotransduction pathways, which interfere with insulin signaling leading to insulin resistance. Dysfunctional adipose tissue in obesity secretes several circulating serpins, which lead to systemic effects, including endothelial dysfunction, thrombosis, hepatic insulin resistance, and impaired peripheral glucose uptake. These interconnected mechanisms establish a pathogenic axis linking serpins and adipose tissue to systemic cardiometabolic disease. Strategies for targeting pro-inflammatory serpins may offer therapeutic opportunities, and circulating serpin levels may be used as biomarkers for disease progression and assessment of cardiometabolic risk. Created with https://www.biorender.com accessed on 8 April 2026.

Insulin resistance is not confined to adipose tissue; it represents a systemic state affecting multiple organs, including the liver and skeletal muscle. Adipokines and inflammatory mediators released from dysfunctional adipose tissue serve as key communicators in this inter-organ crosstalk (Figure 4).

Elevated levels of PAI-1 are a feature of metabolic syndrome and are associated with insulin resistance in the liver and muscle [202,203]. In the liver, elevated PAI-1 correlates with fibrosis and insulin resistance and directly regulates hepatic lipid metabolism by controlling genes like Pcsk9 [204,209]. Similarly, PEDF, which is elevated in obesity, has been shown to impair insulin-mediated whole-body glucose uptake, with a particular effect on the liver, where it increases hepatic gluconeogenesis, indicative of hepatic insulin resistance [6]. On the other hand, central administration of vaspin in high-fat-diet-fed rats was found to inhibit hepatic glucose production, thereby improving hepatic insulin sensitivity. Interestingly, this effect was mediated by the vagus nerve, demonstrating a direct neural link between the central nervous system and hepatic glucose metabolism [77]. Vaspin also influences satiety, as central administration reduces food intake in rodents through decreased expression of orexigenic neuropeptide Y (NPY) and increased expression of anorexigenic proopiomelanocortin (POMC) in the hypothalamus [78]. This highlights an additional dimension of serpin function, extending beyond peripheral metabolic tissues to central regulation of energy balance.

A critical novel player in this inter-organ axis is SerpinA3k (the mouse homolog of human SerpinA3). SerpinA3k deficiency in a murine model of type 2 diabetes (T2D) was shown to provide significant metabolic protection. Diabetic SerpinA3k-deficient mice displayed improved glycemic control, higher pancreatic insulin content, reduced insulin resistance, and attenuated renal hyperfiltration compared to diabetic wild-type mice [210]. This was associated with a favorable remodeling of visceral adipocytes, characterized by a higher proportion of small, insulin-sensitive adipocytes and preserved lipolytic function [210]. These findings suggest that targeting SerpinA3k may offer a novel therapeutic strategy for preserving adipose tissue function, improving insulin sensitivity, and thereby mitigating the metabolic complications of obesity and type 2 diabetes.

The systemic nature of insulin resistance is driven by inter-organ communication. Serpins like PAI-1 and PEDF, secreted from dysfunctional adipose tissue, directly impair insulin signaling in the liver, while protective serpins like vaspin can improve hepatic insulin sensitivity and decrease appetite. Novel regulators such as SerpinA3k further illustrate how serpins impact adipose tissue remodeling and metabolic health.

### 8.5. Depot-Specific and Systemic Perspectives

Adipose tissue heterogeneity adds another layer of complexity to insulin sensitivity. Different adipose depots exhibit distinct serpin expression profiles, which shape their metabolic impact. Therefore, elucidating the functional differences between these depots is critical to understand their implications for metabolic health.

Visceral adipose tissue (VAT) is considered metabolically pathogenic. Its accumulation is a key determinant of metabolic disorders such as insulin resistance, type 2 diabetes, and atherosclerosis [211,212]. VAT is more inflammatory and releases a different profile of adipokines compared to subcutaneous fat. For instance, PAI-1 is synthesized by adipocytes, particularly in human VAT, and elevated PAI-1 levels have been linked to metabolic syndrome [201]. Similarly, the pathogenic serpin PEDF has been linked to factors related to visceral obesity, including waist circumference and triglyceride levels [79]. Moreover, gene expression studies confirm that VAT and subcutaneous fat are biologically distinct, with differential expression of genes involved in metabolic homeostasis, including a higher expression of PAI-1 in VAT [213]. This depot-specific enrichment of pathogenic serpins explains, in part, why VAT is closely linked to increased cardiometabolic risk. On the other hand, subcutaneous adipose tissue (SAT) is generally considered metabolically protective. While vaspin mRNA is expressed in both VAT and SAT in obese subjects, multivariate analysis revealed that insulin sensitivity was the strongest predictor of subcutaneous vaspin expression [69]. Thus, SAT may act as a reservoir of protective serpins that buffer systemic metabolic stress.

BAT further expands this paradigm. Unlike WAT, which primarily stores energy, BAT dissipates energy as heat through the activity of mitochondrial uncoupling protein-1 (UCP1). This thermogenic activity increases energy expenditure and is associated with improved systemic insulin sensitivity [214]. BAT is now recognized as a metabolically dynamic and multifunctional tissue that contributes to glucose, lipid, and branched-chain amino acid homeostasis and secretes batokines that enhance insulin sensitivity and exert anti-inflammatory effects [215]. Importantly, vaspin expression is specifically induced in BAT after obesogenic diet consumption or cold exposure, with vaspin mRNA levels increasing up to 10-fold in BAT of high-fat- or high-sugar-fed mice and 5-fold in cold-exposed mice, suggesting a functional role for vaspin in BAT activation and thermogenesis [65]. This suggests that serpins may play a role in mediating some of BAT’s protective functions, thereby linking thermogenesis to insulin sensitivity and overall metabolic health.

In summary, the depot-specific nature of adipose tissue function is a critical determinant of metabolic health. Visceral fat is linked to the secretion of pathogenic serpins like PAI-1 and PEDF and insulin resistance, whereas subcutaneous fat may be a source of compensatory, insulin-sensitizing serpins like vaspin. The emerging role of brown fat, with its increased vaspin expression, adds another layer of complexity to the depot-specific nature of adipose tissue and the role of serpins in regulating metabolic homeostasis.

## 9. Emerging Technologies and Future Directions

The integration of single-cell RNA sequencing (scRNA-seq) with traditional transcriptomic workflows is fundamentally reshaping the study of the Serpin family by resolving cellular heterogeneity and identifying precise niches of action [216,217]. ScRNA-seq enables precise mapping of serpin expression across adipose-resident cell populations, including adipocytes, macrophages, endothelial cells, and fibroblasts, revealing cell type-specific roles in inflammation, extracellular matrix remodeling, and metabolic regulation [218]. Complementary spatial transcriptomics further localizes serpin activity within distinct adipose niches, such as fibrotic, hypoxic, or immune-enriched regions, providing insight into how protease–antiprotease balance is organized in situ [219].

Several landmark single-cell RNA sequencing (scRNA-seq) and single-nucleus RNA sequencing (snRNA-seq) studies have mapped the cellular landscape of human white adipose tissue (WAT), providing a framework for understanding depot-specific serpin biology. Vijay et al. [220] identified distinct immune, endothelial, fibroblast, and adipocyte progenitor populations within subcutaneous SAT and visceral adipose tissue VAT, revealing depot-specific transcriptional programs associated with extracellular matrix remodeling and protease–antiprotease regulation [220]. Building on these findings, Emont et al. [221] generated integrated human and mouse adipose atlases that resolved adipose stem and progenitor cell subtypes, vascular cells, immune cells, and mature adipocytes, highlighting marked depot-specific cellular heterogeneity that may influence serpin-associated regulation of adipogenesis, fibrosis, and metabolic function [221].

More recently, Lazarescu et al. [222] identified adipocyte subpopulations specialized in immune signaling, extracellular matrix deposition, vascularization, and mitochondrial metabolism, suggesting that serpin expression may vary across adipocyte differentiation states and adipose depots [222]. Complementary studies by Hepler et al. [223] further defined functionally distinct stromal populations within visceral adipose tissue, including adipogenic progenitors and fibro-inflammatory progenitors that regulate adipose remodeling and inflammation [223].

Multi-omics approaches have extended these observations by linking serpin expression to adipose tissue dysfunction. Quantitative proteomic analyses identified depot-specific serpin signatures associated with ECM remodeling and inflammation [224], whereas clinical studies demonstrated associations between circulating SERPINE1 (PAI-1) and cardiometabolic risk markers [225].

Computational cell–cell communication analysis tools, such as CellChat, have emerged as powerful approaches for investigating serpin-associated signaling within complex tissue microenvironments. Wang et al. [226] applied CellChat to a bovine skeletal muscle single-cell atlas and identified extensive communication networks between vascular cells and fibro/adipogenic progenitors, including pathways involving serpin family ligands [226]. Although these findings were not derived from adipose tissue, they demonstrate the utility of ligand–receptor modeling for resolving serpin-mediated intercellular interactions [226]. Similar approaches could be applied to human adipose tissue to systematically investigate adipocyte–immune and stromal cell crosstalk, providing insights into how serpins influence macrophage polarization, T-cell recruitment, extracellular matrix remodeling, and cytokine-mediated inflammatory responses during obesity.

Spatial transcriptomics (ST) complements single-cell approaches by preserving the spatial organization of adipose tissue and its cellular interactions. Using the Visium platform, Bäckdahl et al. [227] identified three spatially distinct adipocyte subpopulations in human subcutaneous adipose tissue (SAT) with differential insulin responsiveness, highlighting adipocyte heterogeneity [227]. Similarly, Kohda et al. [142] showed that Clec4e+ macrophages localize to regions of extracellular matrix remodeling and crown-like structure formation in murine epididymal white adipose tissue during high-fat feeding [142]. Although direct spatial analyses of serpin expression in human adipose tissue remain limited, ST provides a promising framework for investigating the spatial roles of serpins in adipose remodeling, inflammation, and metabolic regulation during obesity [219].

Integration of these datasets within emerging human adipose tissue atlases and multi-omics platforms provides an opportunity to link serpin expression with clinical phenotypes and metabolic outcomes. The use of integrative multi-omics and machine learning algorithms to identify robust serpin biomarkers is an emerging approach. Methods such as LASSO, SVM-RFE, and Boruta have been successfully applied to prioritize candidate serpin biomarkers and regulatory networks, facilitating the identification of key serpin-associated pathways and potential therapeutic targets [216,228]. Furthermore, computational tools such as CellChat enable the mapping of complex intercellular communication networks, providing insights into how serpin-mediated signaling influences adipocyte, immune, and stromal cell interactions within the adipose microenvironment [229].

Future directions in serpin research are increasingly focused on clinical translation and rational drug development. Integrative single-cell and multi-omics approaches may facilitate the development of predictive models that combine serpin expression profiles with clinical and metabolic features to improve disease stratification and therapeutic decision-making [216,217,228]. In parallel, structure-based molecular docking and drug repurposing strategies are being explored to identify existing compounds capable of modulating serpin activity; for example, Lenvatinib and Dasatinib have been proposed as potential SERPINE1 inhibitors [228]. As research progresses, integrating these emerging technologies with targeted modulation of serpin-associated signaling pathways, including WNT and ERK1/2 networks, may provide novel opportunities to improve our understanding of adipose remodeling and to develop precision therapies for obesity and related metabolic disorders [216,217,228,229,230].

## 10. Conclusions

Serpins are central regulators of adipose tissue biology, linking ECM remodeling, inflammation, and insulin signaling. In obesity, dysregulated serpin expression contributes to fibrosis, immune activation, and metabolic dysfunction, whereas protective serpins help preserve adipose tissue plasticity and insulin sensitivity. Their depot-specific expression patterns and responsiveness to hypoxic and inflammatory cues underscore their dynamic and context-dependent roles in adipose tissue remodeling and disease progression.

However, current evidence is largely associative, with limited functional validation in human adipose tissue. Redundancy within the serpin family and variability across experimental models further complicate the interpretation of individual serpin functions and their translational relevance.

Future research should prioritize high-resolution, human-centered approaches, including single-cell and spatial transcriptomics, depot-resolved profiling, and adipocyte–immune interaction mapping integrated with human adipose tissue atlases. These strategies will be essential for defining causal mechanisms, identifying adipose-specific serpin signatures, clarifying the context-dependent roles of individual serpins, and facilitating the development of novel therapeutic targets for obesity and metabolic disease.

## Data Availability

Not applicable. This is a review article, and all relevant information is provided within the article.
